# Outcome of right ventricular assist device implantation following left ventricular assist device implantation: Systematic review and meta-analysis

**DOI:** 10.1177/02676591211024817

**Published:** 2021-06-11

**Authors:** Gregory Reid, Constantin Mork, Brigita Gahl, Christian Appenzeller-Herzog, Ludwig K von Segesser, Friedrich Eckstein, Denis A Berdajs

**Affiliations:** 1 Department of Cardiac Surgery, University Hospital Basel, Basel, Switzerland; 2 University Medical Library, University Library of Basel, Basel, Switzerland; 3 Department of Surgery and Anesthesiology Cardio-Vascular Research, University Hospital Lausanne, CHUV, Switzerland

**Keywords:** left ventricular assist device, right ventricular assist device, right heart failure

## Abstract

**Objectives:**

The main aim was a systematic evaluation of the current evidence on outcomes for patients undergoing right ventricular assist device (RVAD) implantation following left ventricular assist device (LVAD) implantation.

**Methods:**

This systematic review was registered on PROSPERO (CRD42019130131). Reports evaluating in-hospital as well as follow-up outcome in LVAD and LVAD/RVAD implantation were identified through Ovid Medline, Web of Science and EMBASE. The primary endpoint was mortality at the hospital stay and at follow-up. Pooled incidence of defined endpoints was calculated by using random effects models.

**Results:**

A total of 35 retrospective studies that included 3260 patients were analyzed. 30 days mortality was in favour of isolated LVAD implantation 6.74% (1.98–11.5%) versus 31.9% (19.78–44.02%) p = 0.001 in LVAD with temporary need for RVAD. During the hospital stay the incidence of major bleeding was 18.7% (18.2–19.4%) versus 40.0% (36.3–48.8%) and stroke rate was 5.6% (5.4–5.8%) versus 20.9% (16.8–28.3%) and was in favour of isolated LVAD implantation. Mortality reported at short-term as well at long-term was 19.66% (CI 15.73–23.59%) and 33.90% (CI 8.84–59.96%) in LVAD respectively versus 45.35% (CI 35.31–55.4%) p ⩽ 0.001 and 48.23% (CI 16.01–80.45%) p = 0.686 in LVAD/RVAD group respectively.

**Conclusion:**

Implantation of a temporary RVAD is allied with a worse outcome during the primary hospitalization and at follow-up. Compared to isolated LVAD support, biventricular mechanical circulatory support leads to an elevated mortality and higher incidence of adverse events such as bleeding and stroke.

## Introduction

Implantable mechanical circulatory devices, such as left ventricular assist device (LVAD), have emerged as a relevant option for improving quality of life and survival in patients with end-stage heart failure and are commonly utilized. The most common indications include bridge to transplant, bridge to candidacy, destination therapy and bridge to recovery.^1^ Technological developments have led to the use of continuous flow devices, which are superior to previous pulsatile models with regard to efficiency, size, implantability, extended support and overall patient outcomes. Results of clinical practice have led to an expanded role of LVAD in clinical use.^2–4^ LVAD implantation improves exercise tolerance and end-organ dysfunction and can improve hemodynamics.^5^

However, despite the excellent results, early right heart failure (RHF) or a progressive decline of right ventricular function remain major problems. Right heart failure may lead to impaired LVAD flow, difficulty in weaning from cardio-pulmonary bypass (CPB), decreased tissue perfusion, prolonged inotropic support and multi-organ failure, all associated with increased morbidity and mortality.^6^ The incidence of patients with right ventricular failure following LVAD implantation ranges between 9 and 44%^1,3,7^ and about 10%–15% of these patients may require subsequent implantation of a separate right ventricular assist device (RVAD), associated with a higher mortality.^1,8,9^

Patients requiring RVAD support represent a heterogeneous group ranging from patients in cardiogenic shock after a myocardial infarction to those with a known history of chronic heart failure. Additionally, it seems that timing of RVAD implantation may play a crucial role in in-hospital as well in short-term survival.^1^

The main aim of this systematic review was to synthesize all available evidence on the implantation of a RVAD in a LVAD setting and contribute to a better understanding of long-term outcomes following this surgical intervention. A systematic review summarizes the research evidence on a subject of interest and provides a more objective insight at the research level, compared to other study methods such as narrative reviews and expert commentaries.

## Material and methods

This systematic review follows the recommendations for preferred reporting items in systematic reviews and the meta-analyses (PRISMA) statement on conducting systematic reviews of RCTs.^10^ It was prospectively registered on PROSPERO (CRD42019130131).

### Search strategy

An information specialist (C.A.-H.) developed the search strategy, which was internally peer-reviewed by a second information specialist. Text words (synonyms and word variations) and database-specific subject headings for ventricular assist devices, right ventricular failure and ‘left and right’ were used. The detailed search strategies for Ovid Medline, Web of Science and EMBASE can be found in Appendix 1 (Supplemental material). Search results were exported to EndNote X9 and deduplicated. Articles in English, German and French were considered for analysis by two reviewers (C.M. and G.R.) who screened the titles and abstracts of identified records and subsequently, the full texts of selected abstracts. An arbitrary reviewer (D.B.) assessed whether inclusion and exclusion were performed correctly. In cases of disagreement, an agreement was negotiated. References of selected articles were crosschecked for other relevant studies. Authors were contacted when a publication could not be obtained or not all required information could be retrieved from the publication.

### Inclusion/exclusion criteria

The analysis did not include laboratory experimental reports, animal studies, studies on a paediatric population, in vitro studies, editorials, letters and case reports, as well reports on other clinical results. Criteria for inclusion were reports on adults, articles reporting on mortality/survival and/or morbidity after LVAD and/or after a LVAD/RVAD procedure, minimal duration of follow-up ⩾1 year, completeness of follow-up 90% (high quality) and study size *n* ⩾ 10. In a case of multiple publications on the same patient cohort, the most recent publication was included in the analysis.

### Quality assessment

The internal validity of each study was assessed using the Scottish Intercollegiate Guidelines Network (SIGN) Methodology checklist for RCTs by two independent reviewers (C.M. and R.G.).^11^

Quality was rated as follows: ‘High (+ +)’ denoted that most of the criteria were fulfilled. If not fulfilled, the conclusions of the study are very unlikely to alter. ‘Moderate (+)’ denoted that some criteria were fulfilled. Criteria not adequately described are unlikely to alter the conclusions. ‘Low (−)’ denoted that few or no criteria were fulfilled and the conclusions are likely to alter.

### Data extraction

Microsoft Excel and Review Manager version 4.2 for Windows (The Cochrane Collaboration, 2003) were used for data extraction. To control for potential heterogeneity caused by the procedure, publications were allocated to the following categories: (1) series on LVAD and; (2) series on LVAD and RVAD, respectively. Study design was documented in each paper entering inclusion criteria. Data with regard to the authorship, date of publication, cohort quantity (sample size, gender, age), genesis of heart failure, study design, follow-up duration as well the defined endpoint were extracted. Outcome events were registered according to the 2008 American Association for Thoracic Surgery/Society of Thoracic Surgeons/European Association for Cardiothoracic Surgery guidelines.^12^

### Primary and secondary endpoints

Primary endpoints were defined as: in-hospital mortality (30 days), mortality/survival at 1 year (short-term), 3 years (mid-term) and 5 years (long-term) of follow-up. Secondary endpoints were: stroke, thromboembolic event, bleeding and transplantation rate.

### Statistical analysis

We calculated median and IQR of reported patient characteristics at baseline per treatment group (LVAD vs LVAD/RVAD), to approximately address the question of homogeneous indication criteria for either treatment. In the case of papers not reporting the mean, we included the median as an approximation. If standard deviation was not reported, we replaced it by the mean of all reported standard deviations. We calculated pooled Kaplan–Meier estimates of survival at 30 days, 1 year, 3 and 5 years using random effects models. Corresponding confidence intervals were calculated using Wilsons method based on number of patients at risk if reported. In studies that did not report patients at risk but only Kaplan–Meier estimates, we imputed the mean standard error to approximate confidence intervals. We assumed no patients lost to follow-up within the first 30 days. We used meta-regression with random effects to address heterogeneity, including time of publication as covariate in addition to treatment, first as before or during 2014 versus later, then as year of publication.

We calculated pooled rates per 100 patient years and standard errors for transplantation by using random effects models on log transformed rates per year. We back-transformed the rates and corresponding limits and derived rates per 100 patient-years. If cumulated years of follow-up was not reported, we approximated it by multiplying the number of patients by the mean follow-up time. All other secondary outcomes were reported less exhaustively in the original publications. Therefore, we refrained from calculating pooled rates of complications, but summarized these variables as medians and IQRs for LVAD procedure and LVAD/RVAD procedure separately. Analyses were performed using Stata version 16 (StataCorp, College Station, TX, USA).

## Results

**Figure 1.**
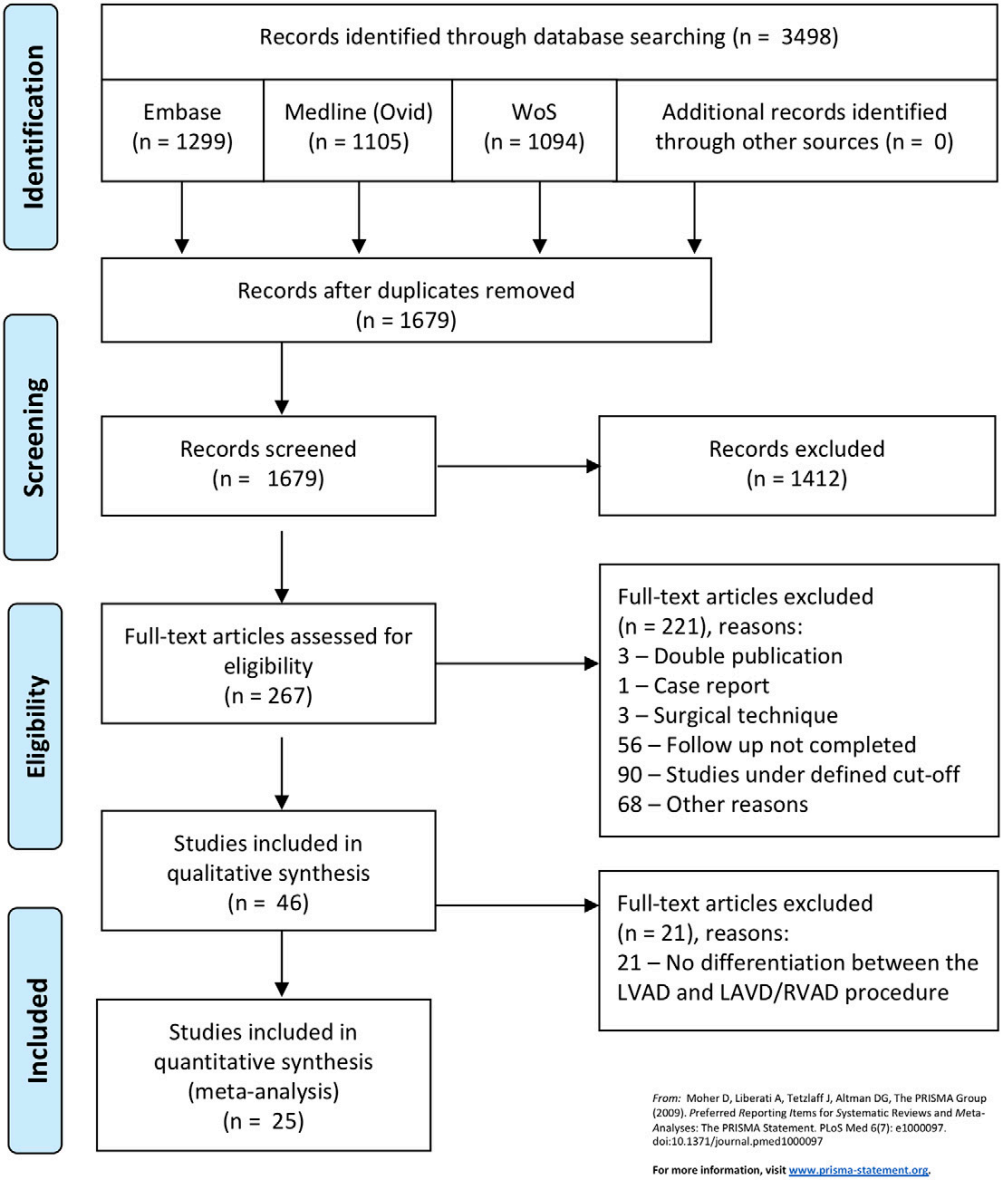
Flow sheath of literature extraction strategy.

Our searches returned 1679 unique records and of these, 267 abstracts were chosen for a final reading. A total of 242 full-text records were excluded for the following reasons: no differentiation between the LVAD and LVAD/RVAD procedure (*n* = 21), double publication (*n* = 3), case report (*n* = 1), surgical technique (*n* = 3), follow-up not completed (*n* = 56), studies under defined cut-off (*n* = 90) and other (*n* = 68) (Figure 1).

Finally, 25 retrospective studies including 3260 patients published between 1991 and 2020 met our inclusion criteria. No additional relevant records were identified in the reference lists of these articles. Overall, 10 comparator (two-arm) studies were analysed, along with a further nine studies dealing with the LVAD procedure and six studies with the LVAD/RVAD procedure. The meta regressions showed a high heterogeneity among studies with respect to 1-year survival, with a high *I*^2^.

Median patient number per study arm was 98 (IQR 56–241) for LVAD and 27 (IQR 16–37.5) for the LVAD/RVAD procedure. Supplemental material Table 1 provides an overview of the publications considered for analysis, 10 reports compared both surgical procedures, with *n* = 1962 patients in LVAD and with *n* = 469 patients in the LVAD/RVAD group, nine series evaluated the outcome only on the LVAD procedure (*n* = 829 patients) and five reports were on LVAD and permanent RVAD procedure (*n* = 182 patients).

The SIGN checklist (supplementary material available online with this article) was used to assess risk of bias within studies. The study quality was good in all studies.

Descriptive statistics are presented in Table 1, including preoperative data, indication for the interventions and the reported INTERMACS class. There is a trend towards a higher central venous pressure (CVP), lower mean pulmonary artery pressure (mPAP) and higher incidence of sever tricuspid valve insufficiency (TVI) in the LVAD/RAVD group. These characteristics are coupled with a high likelihood to be on preoperative mechanical support, when compared to the LVAD only group. Studies reporting on both treatment modalities are presented in Supplemental Table 2. Patients who underwent LVAD/RVAD implantation were found to be 3.5 years younger than patients who received LVAD alone. INTERMACS classification 1 or 2 was slightly more frequent in patients who received both, LVAD/RVAD, not reaching significance.

**Table 1.** Summary of population characteristics.

Study characteristics	LVAD	LVAD/RVAD
Mean age of patient at intervention (years)		
Median (IQR)	51.5 (48.0–55.0)	51.0 (49.9–52.2)
Range	38.0–60.8	39.6–56.2
Reported in (*n*/total)	18/19	14/16
Number of patients per study arm (*n*)		
Median (IQR)	98 (56–241)	27 (16–37.1)
Range	12–454	11–379
Reported in (*n*/total)	19/19	16/16
Female (%)		
Median (IQR)	18.4 (13.6–20.3)	26.3 (17.6–35.0)
Range	4.4–53.6	9.1–37.0
Reported in (*n*/total)	17/19	14/16
Diabetes (%)		
Median (IQR)	27.6 (15.6–35.3)	35.4 (29.6–40.0)
Range	9.4–42.7	8.6–40.0
Reported in (*n*/total)	9/19	6/16
COPD (%)		
Median (IQR)	9.9 (4.2–15.1)	6.7 (0.0–13.3)
Range	1.7–17.0	0.0–13.3
Reported in (*n*/total)	4/19	2/16
ECMO prior LVAD implantation (%)		
Median (IQR)	9.3 (7.7–11.2)	20.9 (17.1–40.7)
Range	7.7–11.2	3.0–43.3
Reported in (*n*/total)	3/19	5/16
IABP prior LVAD implantation (%)		
Median (IQR)	28.4 (13.6–40.7)	37.0 (28.2–50.0)
Range	12.5–78.6	22.9–60.0
Reported in (*n*/total)	10/19	9/16
Severe tricuspid regurgitation (%)		
Median (IQR)	22.0 (8.6–38.7)	30.7 (11.4–50.0)
Range	8.5–42.1	11.4–50.0
Reported in (*n*/total)	4/19	2/16
Mean pulmonary pressure MPAP (mmHg)		
Median (IQR)	35.3 (32.7–38.0)	33.0 (29.7–37.1)
Range	29.8–38.9	14.5–37.8
Reported in (*n*/total)	15/19	9/16
Right atrial pressure RAP (mmHg)		
Median (IQR)	12.1 (11.6–14.3)	15.3 (15.0–17.9)
Range	9.5–15.0	9.9–25.0
Reported in (*n*/total)	7/19	5/16
Central venous pressure CVP mean (mmHg)		
Median (IQR)	11.5 (11.1–13.2)	14.2 (10.7–16.1)
Range	9.3–20.8	7.5–26.3
Reported in (*n*/total)	9/19	7/16
Ischaemic cardiomyopathy (%)		
Median (IQR)	34.6 (21.4–46.7)	50.0 (26.7–55.6)
Range	9.4–64.4	8.5–207.4
Reported in (*n*/total)	15/19	13/16
Dilated cardiomyopathy (%)		
Median (IQR)	70.7 (64.3–84.6)	62.0 (45.5–80.0)
Range	64.3–84.6	45.5–80.0
Reported in (*n*/total)	3/19	3/16
BUN mean (mg/dl)		
Median (IQR)	30.0 (25.6–32.0)	32.8 (29.5–38.5)
Range	9.4–37.0	8.7–78.2
Reported in (*n*/total)	11/19	8/16
Bilirubin mean (mg/dl)		
Median (IQR)	1.4 (1.2–1.7)	1.9 (1.6–2.2)
Range	0.9–3.3	1.4–4.2
Reported in (*n*/total)	15/19	9/16
ASAT mean (U/L or IU/L)		
Median (IQR)	75.4 (46.0–83.7)	60.7 (37.5–169.0)
Range	10.3–181.5	37.5–169.0
Reported in (*n*/total)	9/19	3/16
Mean duration hospital stay after surgery (days)		
Median (IQR)	33.9 (33.9–33.9)	
Range	33.9–33.9	
Reported in (*n*/total)	1/19	
INTERMACS class I and II (%)		
Median (IQR)	56.0 (49.1–67.9)	66.2 (44.0–71.4)
Range	45.1–86.7	43.7–86.7
Reported in (*n*/total)	7/19	6/16
INTERMACS class III and IV (%)		
Median (IQR)	25.0 (13.3–40.2)	31.0 (25.7–54.9)
Range	4.7–44.0	13.3–56.0
Reported in (*n*/total)	6/19	6/16
INTERMACS class > V (%)		
Median (IQR)	7.1 (2.1–9.4)	8.6 (8.6–8.6)
Range	2.1–9.4	8.6–8.6
Reported in (*n*/total)	3/19	1/16

**Figure 2.**
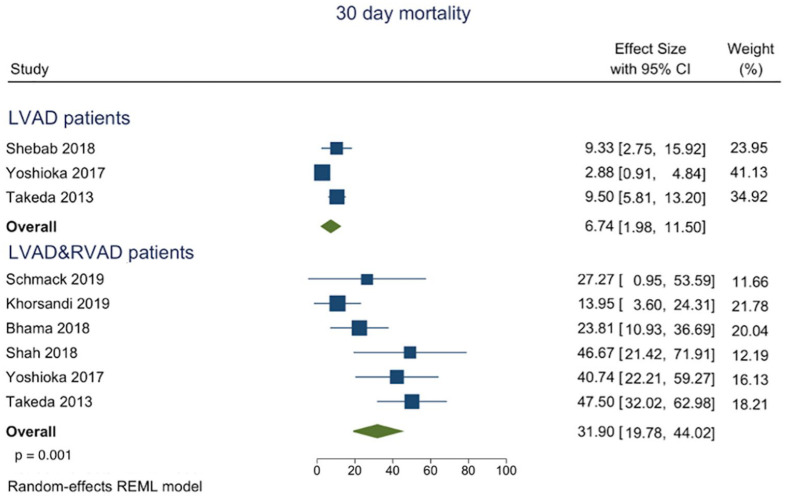
Forest plot of proportions of 30 days mortality in regard to the intervention modality.

**Figure 3.**
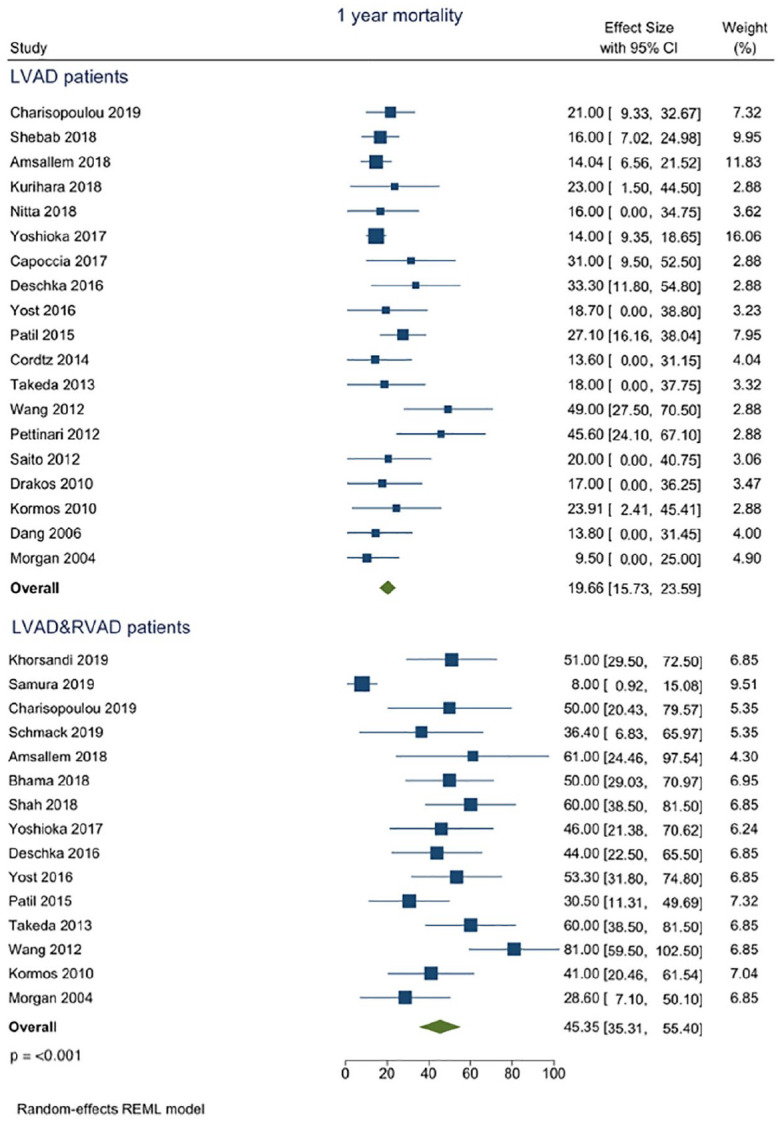
Forest plot of proportions of mortality at the short-term in regard to the intervention modality.

Pooled in-hospital mortality was 6.74% (95% CI 1.98–11.50) in LVAD versus 31.9% (95% CI 19.78–44.02) p = 0.001 in the LVAD/RVAD cohort (Figure 2). One-year mortality was also in favour of isolated LVAD implantation (Figure 3) with 19.66% (95% CI 15.73–23.59) in LVAD versus 45.35% (95% CI 35.31–55.40) p ⩽ 0.001 in the LVAD/RVAD group (Figure 3).

**Figure 4.**
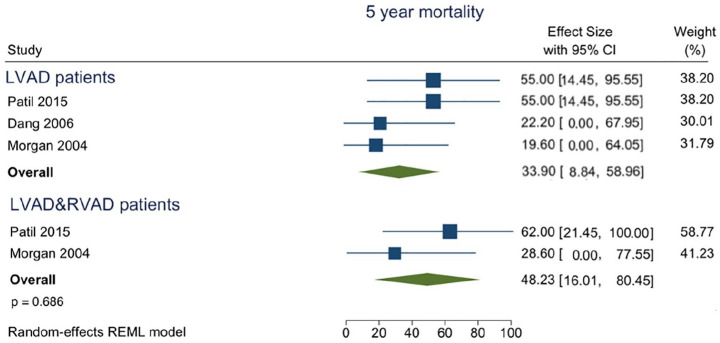
Forest plot of proportions of mortality at the long-term in regard to the intervention modality.

Five studies with 461 patients at risk reported mortality with a follow-up of 5 years (Figure 4), three studies were on the LVAD procedure (*N* = 409) with a pooled mortality estimate of 33.90% (95% CI 8.84–58.96%) and two studies were on the LVAD/RVAD procedure (*N* = 52) with a pooled mortality estimate of 48.23% (95% CI 16.01–80.45%) p = 0.686. As CIs were very large in all studies, due to small numbers of patients at risk, heterogeneity *I*^2^ was small (Table 2).

**Table 2.** Primary endpoints in in-hospital, short- and mid-term follow-up by procedure.

In-hospital mortality (30 days) (%)			
Author		Author	
LVAD		LVAD/RVAD	
Shehab et al.^16^	9.3 (4.6–18.0)	Schmack et al.^41^	27.3 (9.7–56.6)
Yoshioka et al.^15^	2.9 (1.5–5.6)	Khorsandi et al.^17^	14.0 (6.6–27.3)
Takeda et al.^14^	9.5 (6.4–13.9)	Bhama et al.^40^	23.8 (13.5–38.5)
Charisopoulou et al.^28^	nr	Shah et al.^42^	46.7 (24.8–69.9)
Amsallem et al.^27^	nr	Yoshioka et al.^15^	40.7 (24.5–59.3)
Kurihara et al.^33^	nr	Takeda et al.^14^	47.5 (32.9–62.5)
Nitta et al.^31^	nr	Samura et al.^21^	nr
Capoccia et al.^44^	nr	Charisopoulou et al.^28^	nr
Deschka et al.^29^	nr	Amsallemet al.^27^	nr
Yost et al.^32^	nr	Deschka et al.^29^	nr
Patil et al.^18^	nr	Yost et al.^32^	nr
Cordtz et al.^43^	nr	Patil et al.^18^	nr
Wang et al.^24^	nr	Wang et al.^24^	nr
Pettinari et al.^34^	nr	Kormos et al.^1^	nr
Saito et al.^35^	nr	Morgan et al.^9^	nr
Drakos et al.^30^	nr		
Kormos et al.^1^	nr		
Dang et al.^19^	nr		
Morgan et al.^9^	nr		
Pooled estimate (95% CI)	6.47 (1.98–11.50)	Pooled estimate (95% CI)	31.9 (19.78–44.02)
	*p* = 0.001		
1-year mortality (%)			
Author		Author	
LVAD		LVAD/RVAD	
Shehab et al.^16^	16.0 (8.9–26.8)	Schmack et al.^41^	36.4 (15.8–75.0)
Yoshioka et al.^15^	14.0 (10.0–19.3)	Khorsandi et al.^17^	51.0 (29.5–72.5)
Takeda et al.^14^	18.0 (0.0–39.5)	Bhama et al.^40^	50.0 (29.0–71.0)
Charisopoulou et al.^28^	21.0 (11.2–34.5)	Shah et al.^42^	60.0 (38.5–81.5)
Amsallem et al.^27^	14.0 (8.4–23.3)	Yoshioka et al.^15^	46.0 (25.4–74.6)
Kurihara et al.^33^	23.0 (1.5–44.5)	Takeda et al.^14^	60.0 (38.5–81.5)
Nitta et al.^31^	16.0 (0.0–37.5)	Samura et al.^21^	8.0 (2.8–17.0)
Capoccia et al.^44^	31.0 (9.5–52.5)	Charisopoulou et al.^28^	50.0 (15.8–75.0)
Deschka et al.^29^	33.3 (11.8–54.8)	Amsallem et al.^27^	61.0 (20.8–93.9)
Yost et al.^32^	18.7 (0.0–40.2)	Deschka et al.^29^	44.0 (22.5–65.5)
Patil et al.^18^	27.1 (17.1–39.0)	Yost et al.^32^	53.3 (31.8–74.8)
Cordtz et al.^43^	13.6 (0.0–35.1)	Patil et al.^18^	30.5 (12.5–50.9)
Wang et al.^24^	49.0 (27.5–70.5)	Wang et al.^24^	81.0 (59.5–102.5)
Pettinari et al.^34^	45.6 (24.1–67.1)	Kormos et al.^1^	41.0 (20.3–61.4)
Saito et al.^35^	20.0 (0.0–41.5)	Morgan et al.^9^	28.6 (7.1–50.1)
Drakos et al.^30^	17.0 (0.0–38.5)		
Kormos et al.^1^	23.9 (2.4–45.4)		
Dang et al.^19^	13.8 (0.0–35.3)		
Morgan et al.^9^	9.5 (0.0–31.0)		
Pooled estimate (95% CI)	19.66 (15.73–23.59)	Pooled estimate (95% CI)	45.35 (35.31–55.4)
	*p* ⩽ 0.001		
5-year mortality (%)			
Author		Author	
LVAD		LVAD/RVAD	
Shehab et al.^16^	nr	Schmack et al.^41^	nr
Yoshioka et al.^15^	nr	Khorsandi et al.^17^	nr
Takeda et al.^14^	nr	Bhama et al.^40^	nr
Charisopoulou et al.^28^	nr	Shah et al.^42^	nr
Amsallem et al.^27^	nr	Yoshioka et al.^15^	nr
Kurihara et al.^33^	nr	Takeda et al.^14^	nr
Nitta et al.^31^	nr	Samura et al.^21^	nr
Capoccia et al.^44^	nr	Charisopoulou et al.^28^	nr
Deschka et al.^29^	nr	Amsallem et al.^27^	nr
Yost et al.^32^	nr	Deschka et al.^29^	nr
Patil et al.^18^	55.0 (14.45–19.55)	Yost et al.^32^	nr
Cordtz et al.^43^	nr	Patil et al.^18^	62.0 (21.45–100.00)
Wang et al.^24^	nr	Wang et al.^24^	nr
Pettinari et al.^34^	nr	Kormos et al.^1^	nr
Saito et al.^35^	nr	Morgan et al.^9^	28.60 (0.00–77.55))
Drakos et al.^30^	nr		
Kormos et al.^1^	nr		
Dang et al.^19^	22.2 (0.00–67.95)		
Morgan et al.^9^	19.6 (0.00–64.05)		
Pooled estimate (95% CI)	33.90 (8.84–58.96)	Pooled estimate (95% CI)	48.23 (16.01–80.45)
	*p* = 0.686		

LVAD: left ventricular assist device; RVAD: right ventricular assist device; nr: not reported.

The meta regressions showed that time of publication did not explain heterogeneity among studies with respect to 1-year survival, as inclusion of time did not decrease *I*^2^. If including the time of publication into the equation, the calculated *I*^2^ of treatment alone (LVAD vs LVAD/RVAD) was 69% for studies before/during 2014; and 86% or 70%, respectively, if >2014 or as year.

Incidence of a thromboembolic event during follow-up was reported in only one study, and was reported as being 40% in the LVAD/RVAD procedure. Incidence of a bleeding event was reported in three reports, one on the LVAD procedure (*n* = 3 patients at risk) and two on the LVAD/RVAD procedure (*n* = 15 patients at risk). After 5 years, the median proportion of patients with a bleeding complication in LVAD was inferior to the proportion in LVAD/RVAD at 7% versus 28.6% (IQR 23.8–33.3%). The proportion of patients that had stroke was 12.2% (IQR 11.1–13.3%) in LVAD versus 21.4% (IQR 5.9–33.3%) in LVAD/RVAD. Transplantation rate was reported in 14 studies, eight were on the LVAD procedure (*n* = 911 patients at risk) and six in the LVAD/RVAD procedure (*n* = 215 patients at risk) (Table 3).

**Table 3.** Secondary endpoints in in-hospital, and mid-/long-term follow-up by procedure.

In-hospital events (%)									
LVAD					LVAD/RVAD				
Author	Neurologic/stroke (%)	Reoperation for bleeding (%)	Thromboembolic event		Author	Neurologic/stroke (%)	Reoperation for bleeding (%)	Thromboembolic event (%)	
Yoshioka et al.^15^	6	13	nr		Yoshioka et al.^15^	19	40	nr	
Takeda et al.^14^	5	21	nr		Takeda et al.^14^	15	57	nr	
Dang et al.^19^	nr	18	nr		Shah et al.^42^	33	nr	13	
Shehab et al.^16^	nr	19	nr		Khorsandi et al.^17^	23	33	nr	
Kormos et al.^1^	nr	19	nr		Kormos et al.^1^	nr	40	nr	
Pettinari et al.^34^	nr	nr	nr		Morgan et al.^9^	nr	nr	nr	
					Bhama et al.^40^	nr	nr	nr	
					Samura et al.^21^	nr	nr	nr	
Overall median % (IQR)	5.6 (5.4–5.8)	18.7 (18.2–19.4)	nr		Overall median % (IQR)	20.9 (16.8–28.3)	40.0 (36.3–48.8)	13.0	
Follow-up events (%)									
LVAD					LVAD/RVAD				
Author	Neurologic/stroke (%)	Thromboembolic event (%)	Bleeding (%)	Transplantation (%)	Author	Neurologic/stroke (%)	Thromboembolic event (%)	Bleeding (%)	Transplantation (%)
Yoshioka et al.^15^	nr	nr	nr	45	Yoshioka et al.^15^	nr	nr	nr	26
Takeda et al.^14^	nr	nr	nr	nr	Takeda et al.^14^	nr	nr	nr	18
Dang et al.^19^	nr	nr	nr	89	Shah et al.^42^	33	40	33	(100)
Shehab et al.^16^	13	nr	nr	61	Khorsandi et al.^17^	nr	nr	nr	nr
Kormos et al.^1^	nr	nr	nr	nr	Kormos et al.^1^	nr	nr	nr	nr
Pettinari et al.^34^	11	nr	7	64	Morgan et al.^9^	6	nr	nr	65
Morgan et al.^9^	nr	nr	nr	72	Bhama et al.^40^	21	nr	24	nr
Patil et l.^18^	nr	nr	nr	18	Patil et al.^18^	nr	nr	nr	20
Saito et al.^35^	nr	nr	nr	25	Samura et al.^21^	nr	nr	nr	8
Deschka et al.^29^	nr	nr	nr	7	Deschka et al.^29^	nr	nr	nr	7
Overall median % (IQR)	12.2 (11.1–13.3)	nr	7.0	53.0 (7.1–89.3)	Overall median % (IQR)	21.4 (5.9–33.3)	40.0	28.6 (23.8–33.3)	20 (8.0–100.0)

LVAD: left ventricular assist device; RVAD: right ventricular assist device; nr: not reported.

Transplantation rates showed large heterogeneity among LVAD studies (*I*^2^ = 97%) and LVAD&RVAD studies (*I*^2^ = 89%). This may emphasize again different indications and approaches at different sites. After LVAD implant, transplantation rate was 31 per 100 patient years (95% CI 20–48) which was slightly higher than after LVAD&RVAD, where the rate was 11 per 100 patient years (CI 4–29), p = 0.055. After excluding the study of Shah 2018 from the LVAD&RVAD group, heterogeneity became small (*I*^2^ < 1%) and the rate dropped to 7 per 100 patient years (CI 5–10), p < 0.001.

## Discussion

This systematic review analyzed the literature focusing on impact of right ventricular failure necessitating RVAD implantation following LVAD for in-hospital and short-term outcome.

LVAD is a well-accepted therapeutic option for patients with end stage heart failure. The incidence of RHF after LVAD implantation is associated with significant perioperative mortality and morbidity ranging between 10 and 40%, a complication that is allied with the elevated mid-term mortality and morbidity.^6–9^

Therapeutic options for severe RHF after LVAD are not evidence-based and are limited to inotropic support or implementation of right ventricular mechanical circulatory support (MCS). Application of permanent biventricular mechanical support (BiVAD) is effective, but is associated with lower survival and reduced quality of life, when compared to isolated LVAD support. Furthermore, the postoperative course is complicated by an elevated incidence of major adverse events. It is however a necessity when conservative treatment by pulmonary vasodilators and inotropes is insufficient.^1,13^ Temporary mechanical circulatory support for right ventricular failure is feasible, reproducible and has been demonstrated to improve outcomes compared with conservative therapy.^8,13^ Reports on mid-term outcome on temporary RVAD after LVAD are, however, limited to a handful of retrospective studies.

The main finding of our analysis is that temporary RVAD implantation following LVAD is associated with decreased in-hospital, as well as short-term survival as compared to isolated LVAD implantation (Figures 2 and 3).^1,14–17^ Similar conclusions regarding the mid-term survival can be drawn, where the implementation of temporary RVAD at index intervention was associated with reduced survival.^9,18–21^ Nevertheless, it seems that the implementation of temporary RVAD at the time of LVAD implantation is associated with a worse outcome.

Our findings conclude that patients requiring temporary RVAD support are more ill at the time of LVAD implantation, compared to those who do not need the support. Indeed, there is evidence that INTERMACS patient profiles were found to be independently associated with temporary RVAD use. Class 1 and 2 were associated with 2–3 times the risk of RVAD compared with profiles > Class 3.^1^ We found a significantly higher number of patients to require preoperative circulatory support by IABP and ECMO in the LVAD/RVAD group.

Besides a ‘general frailty’ of the individual, the hemodynamic performance of the right ventricle at the time of the LVAD implantation as a predictive element for subsequent RHF plays a crucial role. Different metric methods have been developed to assess the risk for RHF. Narrow pulmonary artery pulse pressure (PAPP) is correlated with impaired RV function and is an independent predictive element for RVAD implantation and similar evidence exists for elevated right atrial pressure (RAP).^22–24^ Additionally, the ratio between RAP and PAPP was identified as a risk factor for RVAD.^23,25^ Although the individual variables of the pulmonary artery pulsatility index (RAP and PAPP ratio) were predictors in the INTERMACS model, the index itself did not add incremental value to risk prediction.^26^ Similarly, the RAP/pulmonary capillary wedge pressure (PCWP) ratio, where the index had a significant univariate association with RVAD use which is in contrast to RAP alone, was not an independent predictor.^26^

Regarding hemodynamic variables evaluating pre LVAD right ventricular performance, we found the RAP in the LVAD/RVAD cohort, to be higher when compared to the LVAD cohort.^16,17,24,27–31^ The same may be stated for the central venous pressure, coupled with a lower pulmonary artery pressure.^1,9,14–16,18,19,21,24,27,28,30–35^ The relation between RAP and PAPP (and/or relation to the PCWP) was not evaluated in any of the reports examined. Tricuspid valve insufficiency (TVI) has also been associated with worse outcomes among patients undergoing mechanical circulatory support including RHF.^36,37^ The severity of the TVI is a variable strongly dependent on hemodynamic status and volume load. It may be present in the absence of annular dilatation,^38^ a fact that complicates the liaison between severity of the TVI and RV function. In our analysis, severe TVI was higher in the LVAD/RVAD pooled cohort.^18,27,33,35^ The annulus size of the tricuspid valve, an element that seems to be a more precise indicator of the RV dysfunction and provides an accurate prediction of RHF after LVAD implantation,^38,39^ was not evaluated in the included reports.

Ischaemic cardiomyopathy in our pooled cohorts was higher in LVAD/RVAD as compared to the isolated LVAD group.^1,15,16,18–20,24,27–29,32–35,40–43^ It therefore seems that ischaemic cardiomyopathy is associated with an elevated risk for RVAD implantation. This may be linked to the history of coronary bypass graft surgery, namely it is likely that past cardiac surgery increases the risk of RV injury, prolonged cardiopulmonary bypass time and perioperative bleeding. Elements that may be due to an elevated transfusion rate precipitate the RHF by RV distension and increased pulmonary resistance.

Beside the previously mentioned ‘general frailty’ and worse RV performance at the time of the LVAD implantation, the elevated mortality in the temporary RVAD cohort, may in part be explained by an elevated stroke risk and the need for re-exploration due to bleeding during the hospital stay.^1,14–17,19,42^ A similar conclusion may be drawn for the short- and mid-term period, where the high mortality rate in the temporary RVAD group may in part be explained by an elevated incidence of thromboembolic events, stroke rate and higher bleeding rate in the post discharge period respectively.^9,16,34,40,42^

The literature on mid- and/or long-term survival analysing the outcome of patients requiring a temporary RVAD after LVAD implantation versus isolated LVAD implantations in one single cohort is very limited. We summarized population characteristics of the few studies reporting on both treatments in supplementary Table 1 (supplementary material). Significant differences were found only in the preoperative variables age and Bilirubin serum levels, whereby patients in the LVAD only group were seen to be younger and have lower serum levels, respectively. In-hospital, as well as short-term mortality were significantly higher in temporary RVAD groups. The hemodynamic status of the right ventricle at the time of implantation, augmented biomarkers of end organs as a sign of congestion, as well as a combination of adverse events such as bleeding or thromboembolic events were identified as predictive factors for in-hospital and short-term mortality. In the mid- and long-term mortality analysis, there were very limited studies reporting data. This can be in part due to short follow-up periods in the publications themselves, as well as a non-uniform method of reporting transplantation as an outcome. Furthermore, the studies included were from a variety of healthcare systems.

## Limitations

Timing of RVAD implantation following LVAD was not reported as was not the time frame of the RV failure. Furthermore, the device modalities used as RV circulatory support were not distinguished. The reported devices used varied between nearly all studies and are summarized in supplementary Table 3. We aimed to calculate event rates per 100 patients per year of observation time with standard error within studies, and then derive pooled estimates and corresponding limits of the 95% CI of all primary and secondary outcomes. This was not possible due to incomplete reporting and very small groups. Furthermore, single-arm LVAD studies could not be comprehensively identified. This is due to the search strategy chosen. Other possible limitations include a morbidity bias in the LVAD/RVAD cohorts, as these are inherently prone to be a sicker population associated with a higher mortality.

## Conclusion

Implantation of a temporary RVAD is allied with a worse outcome during the primary hospitalization and at follow up. There is a higher incidence of adverse events such as bleeding and stroke, that may be a functional expression of a dysbalanced hemodynamic status.

## Supplemental Material

sj-pdf-1-prf-10.1177_02676591211024817 – Supplemental material for Outcome of right ventricular assist device implantation following left ventricular assist device implantation: Systematic review and meta-analysis Click here for additional data file. Supplemental material, sj-pdf-1-prf-10.1177_02676591211024817 for Outcome of right ventricular assist device implantation following left ventricular assist device implantation: Systematic review and meta-analysis by Gregory Reid, Constantin Mork, Brigita Gahl, Christian Appenzeller-Herzog, Ludwig K von Segesser, Friedrich Eckstein and Denis A Berdajs in Perfusion

sj-pdf-2-prf-10.1177_02676591211024817 – Supplemental material for Outcome of right ventricular assist device implantation following left ventricular assist device implantation: Systematic review and meta-analysis Click here for additional data file. Supplemental material, sj-pdf-2-prf-10.1177_02676591211024817 for Outcome of right ventricular assist device implantation following left ventricular assist device implantation: Systematic review and meta-analysis by Gregory Reid, Constantin Mork, Brigita Gahl, Christian Appenzeller-Herzog, Ludwig K von Segesser, Friedrich Eckstein and Denis A Berdajs in Perfusion

sj-pdf-3-prf-10.1177_02676591211024817 – Supplemental material for Outcome of right ventricular assist device implantation following left ventricular assist device implantation: Systematic review and meta-analysis Click here for additional data file. Supplemental material, sj-pdf-3-prf-10.1177_02676591211024817 for Outcome of right ventricular assist device implantation following left ventricular assist device implantation: Systematic review and meta-analysis by Gregory Reid, Constantin Mork, Brigita Gahl, Christian Appenzeller-Herzog, Ludwig K von Segesser, Friedrich Eckstein and Denis A Berdajs in Perfusion
