# Supplementary material for: Outcome of right ventricular assist device implantation following left ventricular assist device implantation: Systematic review and meta-analysis
Source: Perfusion. 2021 Jun 11;37(8):773–84. doi: 10.1177/02676591211024817 (PMC9619248; doi:10.1177/02676591211024817)
Supplement: sj-pdf-1-prf-10.1177_02676591211024817 – Supplemental material for Outcome of right ventricular assist device implantation following left ventricular assist device implantation: Systematic review and meta-analysis [file sj-pdf-1-prf-10.1177_02676591211024817.pdf]

Appendix 1: Detailed search strategy

Methods

Eligibility criteria

Criteria for inclusion were publications reporting on mortality and/or morbidity after LVAD and/or LVAD/RVAD procedure, adult patients, minimal duration of follow-up ≥ 1 years, completeness of follow-up ≥ 90%, and study size n ≥ 10. We included all prospective and retrospective studies as well as randomized and non-randomized trials that were written in English, French, or German. In case of multiple publication on the same patient cohort, the most recent publication was included in the analysis. We did not include laboratory experimental reports, studies on a pediatric population, in vitro studies, animal studies, editorials, letters, conference abstracts or other abstract-only publications, case reports or case series, as well as reports on other clinical results.

Identification of relevant literature

An information specialist (C.A.-H.) developed the search strategy, which was internally peer-reviewed by a second information specialist. Text words (synonyms and word variations) and database-specific subject headings for ventricular assist devices, right ventricular failure, and "left and right" were used. We searched the electronic databases: Embase via embase.com, Medline ALL via Ovid, and the Web of Science Core Collection (last search April 21, 2020). No language or date restrictions were applied. All retrieved references were exported to Endnote X9 and deduplicated.

Two reviewers (C.M. and G.R.) screened the references based on their titles and abstracts. Any uncertainties were solved by discussion. All potentially relevant references were retrieved in full-text and independently assessed by two reviewers (C.M and G.R.). Any disagreements over eligibility were resolved by consensus. Where necessary, a third review author (D.B.) made a final judgement.

Results

Our electronic searches identified 1679 records after duplicate removal.

Search strategies

Embase.com

(1299 Results 21.04.2020)

('ventricular assist device'/exp OR 'heart assist device'/de OR 'oxygenator'/exp OR 'blood pump'/exp OR 'pump'/de OR 'assisted circulation'/exp OR (('implant'/de OR 'implantation'/de OR 'devices'/de) AND [<1966-2013]/py) OR (((ventricular OR ventricle OR cardiac OR heart OR vascular OR circulatory) NEAR/5 ('assist device*' OR 'assist system*' OR 'assist unit*' OR assistance OR pump* OR device* OR support)) OR 'artificial ventricle*' OR 'artificial heart ventricle*' OR 'heart ventricle assist*' OR 'assisted circulation' OR VAD OR VAS OR Coapsys OR CorCap OR HeartNet OR 'AB 5000' OR AB5000 OR 'BVS 5000' OR EXCOR OR CentriMag OR Impella OR 'CorAide' OR 'DeBakey Child' OR 'DuraHeart' OR 'EVAHEART' OR 'FlowMaker' OR 'HeartAssist 5' OR 'HeartMate' OR 'HeartQuest' OR 'HeartWare' OR 'INCOR' OR 'Jarvik' OR 'Levacor' OR 'Lion Heart' OR 'LionHeart' OR 'MiTi Heart' OR 'Novacor' OR 'TandemHeart' OR 'Ventrassist' OR 'Protek Duo' OR ((blood OR perfusion) NEAR/3 (pump* OR unit*)) OR oxygenator* OR oxygenation OR oxygenerator* OR 'Affinity NT' OR 'Extracorporeal Life Support*' OR ECMO OR ECLS):ab,ti)

AND

('heart right ventricle failure'/de OR 'heart right ventricle function'/de OR ((right NEAR/3 (heart OR cardiac OR cardinal OR myocardial OR ventricular OR ventricle OR circulatory) NEAR/3 (fail* OR decompensat* OR incompeten* OR insufficien* OR 'stand still' OR dysfunction* OR declin*)) OR (RV NEAR/3 (fail* OR decompensat* OR incompeten* OR insufficien* OR 'stand still' OR dysfunction* OR declin*)) OR (right NEAR/3 HF)):ab,ti)

AND

(((left OR LVAD* OR LVAS OR LV) AND (right OR RVAD* OR RVAS OR RV)):ab,ti)

NOT

('child'/exp NOT 'adult'/exp)

NOT

(('animal'/de OR 'animal experiment'/exp OR 'nonhuman'/de) NOT ('human'/exp OR 'human experiment'/de))

NOT [conference abstract]/lim

Medline (Ovid)

(1105 Results 21.04.2020)

(exp Heart, Artificial/ OR exp oxygenators/ OR Extracorporeal Membrane Oxygenation/ OR assisted circulation/ OR (((ventricular OR ventricle OR cardiac OR heart OR vascular OR circulatory) ADJ5 (assist device* OR assist system* OR assist unit* OR assistance OR pump* OR device* OR support)) OR artificial ventricle* OR artificial heart ventricle* OR heart ventricle assist* OR assisted circulation OR VAD OR VAS OR Coapsys OR CorCap OR HeartNet OR AB 5000 OR AB5000 OR BVS 5000 OR EXCOR OR CentriMag OR Impella OR CorAide OR DeBakey Child OR DuraHeart OR EVAHEART OR FlowMaker OR HeartAssist 5 OR HeartMate OR HeartQuest OR HeartWare OR INCOR OR Jarvik OR Levacor OR Lion Heart OR LionHeart OR MiTi Heart OR Novacor OR TandemHeart OR Ventrassist OR

Protek Duo OR ((blood OR perfusion) ADJ3 (pump* OR unit*)) OR oxygenator* OR oxygenation OR oxygenerator* OR Affinity NT OR Extracorporeal Life Support* OR ECMO OR ECLS).ab,ti.)

AND

(Ventricular Dysfunction, Right/ OR ((right ADJ3 (heart OR cardiac OR cardinal OR myocardial OR ventricular OR ventricle OR circulatory) ADJ3 (fail* OR decompensat* OR incompeten* OR insufficien* OR 'stand still' OR dysfunction* OR declin*)) OR (RV ADJ3 (fail* OR decompensat* OR incompeten* OR insufficien* OR 'stand still' OR dysfunction* OR declin*)) OR (right ADJ3 HF)).ab,ti.)

AND

(((left OR LVAD* OR LVAS OR LV) AND (right OR RVAD* OR RVAS OR RV)).ab,ti.)

NOT

(exp child/ NOT exp adult/)

NOT

(exp animals/ NOT humans/)

Web of Science Core Collection

(1094 Results 21.04.2020)

TS=(((((ventricular OR ventricle OR cardiac OR heart OR vascular OR circulatory) NEAR/5 ("assist device*" OR "assist system*" OR "assist unit*" OR assistance OR pump* OR device* OR support)) OR "artificial ventricle*" OR "artificial heart ventricle*" OR "heart ventricle assist*" OR "assisted circulation" OR VAD OR VAS OR Coapsys OR CorCap OR HeartNet OR "AB 5000" OR AB5000 OR "BVS 5000" OR EXCOR OR CentriMag OR Impella OR "CorAide" OR "DeBakey Child" OR "DuraHeart" OR "EVAHEART" OR "FlowMaker" OR "HeartAssist 5" OR "HeartMate" OR "HeartQuest" OR "HeartWare" OR "INCOR" OR "Jarvik" OR "Levacor" OR "Lion Heart" OR "LionHeart" OR "MiTi Heart" OR "Novacor" OR "TandemHeart" OR "Ventrassist" OR "Protek Duo" OR ((blood OR perfusion) NEAR/2 (pump* OR unit*)) OR oxygenator* OR oxygenation OR oxygenerator* OR "Affinity NT" OR "Extracorporeal Life Support*" OR ECMO OR ECLS)) AND (((right NEAR/2 (heart OR cardiac OR cardinal OR myocardial OR ventricular OR ventricle OR circulatory) NEAR/2 (fail* OR decompensat* OR incompeten* OR insufficien* OR "stand still" OR dysfunction* OR declin*)) OR (RV NEAR/2 (fail* OR decompensat* OR incompeten* OR insufficien* OR "stand still" OR dysfunction* OR declin*)) OR (right NEAR/2 HF))) AND (((left OR LVAD* OR LVAS OR LV) AND (right OR RVAD* OR RVAS OR RV))))

Refined by: [excluding] DOCUMENT TYPES: (MEETING ABSTRACT)
