# Supplementary material for: Outcome of right ventricular assist device implantation following left ventricular assist device implantation: Systematic review and meta-analysis
Source: Perfusion. 2021 Jun 11;37(8):773–84. doi: 10.1177/02676591211024817 (PMC9619248; doi:10.1177/02676591211024817)
Supplement: sj-pdf-2-prf-10.1177_02676591211024817 – Supplemental material for Outcome of right ventricular assist device implantation following left ventricular assist device implantation: Systematic review and meta-analysis [file sj-pdf-2-prf-10.1177_02676591211024817.pdf]

SIGN checklist evaluation protocol for studies included in the analysis

Study	Quality	The study adresses an appropriate and clearly focused question	The two groups being studied are selected from source of population that are comparable in all respects other than the factor under investigation	The study indicates how many of the people asked to take part did so in each groups being studied	The likelihood that some eligible subjects might have the outcome at the time of enrolment is assessed and taken into account in the analysis	What percentage of individuals or clusters recruited into each arm of the study dropped out before the study was completed	Comparison is made between full participants and those lost to follow up, by exposure status	The outcomes are clearly defined	The assessment of outcome is made blind to exposure status. If the study is retrospective this may not be applicable	Where blinding was not possible, there is some recognition that knowledge of exposure status could have influenced the assessment of outcome	The method of assessment of exposure is reliable	Evidence from other sources is used to demonstrate that the method of outcome assessment is valid and reliable	Exposure level or prognostic factor is assessed more than once	The main potential confounders are identified and taken into acount in the design and analysis	Confidence intervals have been provided	How well was the study done to minimise the risk of bias or confounding	Taking into account Clinical considerations, your evaluation of the methodology used, and the statistical power of the study, do you think there is clear evidence of an association between exposure and outcome	Are the results of this study directly applicable to the patient group targeted in this guideline
Amsallem M et al. [27]	++	Y	Y	DNA	DNA	0	DNA	Y	DNA	DNA	Y	Y	Y	Y	Y	++	Y	Y
Capoccia M et al. [44]	++	Y	Y	DNA	DNA	11.8	DNA	Y	DNA	DNA	Y	Y	Y	Y	N	++	N	Y
Charisopoulou et al. [28]	++	Y	Y	DNA	DNA	0	DNA	Y	DNA	DNA	Y	Y	Y	Y	Y	++	Y	Y
Cordtz J et al. [43]	++	Y	Y	DNA	DNA	0	DNA	Y	DNA	DNA	Y	Y	Y	Y	N	++	Y	Y
Dang NC et al. [19]	++	Y	Y	DNA	DNA	0	DNA	Y	DNA	DNA	Y	Y	Y	Y	Y	++	Y	Y
Deschka H et al. [29]	++	Y	Y	DNA	DNA	0	DNA	Y	DNA	DNA	Y	Y	Y	Y	N	++	Y	Y
Drakos SG et al. [30]	++	Y	Y	DNA	DNA	0	DNA	Y	DNA	DNA	Y	Y	N	Y	Y	++	Y	Y
Kormos RL et al. [1]	++	Y	Y	DNA	DNA	0	DNA	Y	DNA	DNA	Y	Y	Y	Y	Y	++	Y	Y
Kurihara C et al. [33]	++	Y	Y	DNA	DNA	0	DNA	Y	DNA	DNA	Y	Y	Y	Y	Y	++	Y	Y
Morgan JA et al. [9]	++	Y	Y	DNA	DNA	0	DNA	Y	DNA	DNA	Y	Y	Y	Y	Y	++	Y	Y
Nitta D et al. [31]	++	Y	Y	DNA	DNA	0	DNA	Y	DNA	DNA	Y	Y	Y	Y	Y	++	Y	Y
Patil NP et al. [18]	++	Y	Y	DNA	DNA	0	DNA	Y	DNA	DNA	Y	Y	Y	Y	Y	++	Y	Y
Pettinari M et al. [34]	++	Y	Y	DNA	DNA	0	DNA	Y	DNA	DNA	Y	Y	Y	Y	N	++	Y	Y
Saito S et al. [35]	++	Y	Y	DNA	DNA	0	DNA	Y	DNA	DNA	Y	Y	Y	Y	Y	++	Y	Y
Shah P et al. [42]	++	Y	Y	DNA	DNA	0	DNA	Y	DNA	DNA	Y	Y	Y	Y	Y	++	Y	Y
Shehab S et al. [16]	++	Y	Y	DNA	DNA	0	DNA	Y	DNA	DNA	Y	Y	Y	Y	Y	++	Y	Y

Takeda K et al. [14]	++	Y	Y	DNA	DNA	0	DNA	Y	DNA	DNA	Y	Y	Y	Y	Y	++	Y	Y
Wang Y et al. [24]	++	Y	Y	DNA	DNA	0	DNA	Y	DNA	DNA	Y	Y	Y	Y	Y	++	Y	Y
Yoshioka D et al. [15]	++	Y	Y	DNA	DNA	0	DNA	Y	DNA	DNA	Y	Y	Y	Y	Y	++	Y	Y
Yost GL et al. [32]	++	Y	Y	DNA	DNA	0	DNA	Y	DNA	DNA	Y	Y	Y	Y	Y	++	Y	Y
Bhama JK et al. [40]	++	Y	Y	DNA	DNA	0	DNA	Y	DNA	DNA	Y	Y	Y	Y	Y	++	Y	Y
Khorsandi M et al. [17]	++	Y	Y	DNA	DNA	0	DNA	Y	DNA	DNA	Y	Y	Y	Y	Y	++	Y	Y
Leidenfrost J et al. [20]	++	Y	Y	DNA	DNA	0	DNA	Y	DNA	DNA	Y	Y	Y	Y	Y	++	Y	Y
Schmack et al. [41]	++	Y	Y	DNA	DNA	0	DNA	Y	DNA	DNA	Y	Y	Y	Y	N	++	Y	Y
Samura et al. [21]	++	Y	Y	DNA	DNA	0	DNA	Y	DNA	DNA	Y	Y	Y	Y	Y	++	Y	Y

Y: yes, N: no, DNA: does not apply, ++; high quality, + moderate quality, - low quality
