# Supplementary material for: Outcome of right ventricular assist device implantation following left ventricular assist device implantation: Systematic review and meta-analysis
Source: Perfusion. 2021 Jun 11;37(8):773–84. doi: 10.1177/02676591211024817 (PMC9619248; doi:10.1177/02676591211024817)

Supplementary Tables

Table 1: Publications overview included in meta-analysis

Study	Year	Procedure	patients (n)	Age mean (years)	Age SD (years)	Female (n)	Female (%)
Takeda K et al. [14]	2013	LVAD	242	55	14	38	15.70
	2013	LVAD/RVAD	379	54.7	13.7	77	20.32
Morgan JA et al. [9]	2004	LVAD	117	45.7	12.9	19	16.24
	2004	LVAD/RVAD	98	56.5	14.4	12	12.24
Patil NP et al. [18]	2015	LVAD	28	50	14.8	15	53.57
	2015	LVAD/RVAD	156	53.3	12.7	22	14.10
Deschka H et al. [29]	2016	LVAD	22			3	13.64
	2016	LVAD/RVAD	45	51.1	13.2	2	4.44
Wang Y et al. [24]	2012	LVAD	76	39.5	16	14	18.42
	2012	LVAD/RVAD	278	58.3	13.4	53	19.06
Yoshioka D et al. [15]	2017	LVAD	40	52	11	14	35.00
	2017	LVAD/RVAD	17	50.4	12.4	3	17.65
Amsallem M et al. [27]	2018	LVAD	35	39.6	14.8	12	34.29
	2018	LVAD/RVAD	25	55.4	12.4	5	20.00
Yost GL et al. [32]	2016	LVAD	15			4	26.67
	2016	LVAD/RVAD	27	56.15	15.06		
Kormos RL et al. [1]	*2010*	LVAD	27	50	15.4	7	25.93
	2010	LVAD/RVAD	30	52.2	14.7	4	13.33
Charisopoulou et al. [28]	2019	LVAD	43			6	13.95
	2019	LVAD/RVAD	14	45	11	5	35.71
Kurihara C et al. [33]	2018	LVAD	66	51	13	4	6.06
Dang NC et al. [19]	2006	LVAD	226	49.6	13.8	43	19.03
Drakos SG et al. [30]	2010	LVAD	56	38	13	13	23.21
Nitta D et al. [31]	2018	LVAD	164	55.98	12.69	40	24.39
Cordtz J et al. [43]	2014	LVAD	75	53	13	15	20.00
Pettinari M et al. [34]	2012	LVAD	454	51.81	13.25	101	22.25
Saito S et al. [35]	2012	LVAD	12	41.16	13.81		
Shehab S et al. [16]	2018	LVAD	27	49.9	10.5	10	37.04
Capoccia M et al. [44]	2017	LVAD	15	50.9	14.4		
Bhama JK et al. [40]	2018	LVAD/RVAD	30	51	13.3	7	23.33
Khorsandi M et al. [17]	2019	LVAD/RVAD	42	55	13	15	35.71
Schmack et al. [41]	2019	LVAD/RVAD	11	51.6	13.1	1	9.09
Shah P et al. [42]	2018	LVAD/RVAD	241	60.8	12.7		
Leidenfrost J et al. [20]	2016	LVAD/RVAD	56	48	12	6	10.71
Samura et al. [21]	2019	LVAD/RVAD	71	46	13	23	32.39

LVAD: left ventricular assist device, RVAD: right ventricular assist device

Table 2: Summary of population characteristics

Variable	Mean difference	Risk difference	p_value
Age (years)	-3.5 (-5.8 to -1.1)		0.004
Female (n)		0.05 (-0.06 to 0.16)	0.336
LVEF (%)	-0.3 (-1.6 to 1.0)		0.619
Right atrial pressure (mmHg)	4.2 (-0.9 to 9.4)		0.107
Bilirubin (mg/dl)	0.7 (0.4 to 1.0)		<0.001
AST (U/l or IU/l)	25.8 (-35.6 to 87.2)		0.411
Central venous pressure (mmHg)	2.4 (0.5 to 4.2)		0.014
MPAP (mmHg)	-3.2 (-6.0 to -0.3)		0.029
Ischemic cardiomyopathy (n)		0.04 (-0.03 to 0.10)	0.278
Diabetes (n)		0.00 (-0.08 to 0.07)	0.920
INTERMACS 1 or 2 (%)		0.08 (-0.10 to 0.27)	0.380

Differences between patients who received LVAD versus LVAD&RVAD in studies reporting on both treatments.

Supplementary Table 3: Summary of LVAD and RVAD models used

LVAD Models:

Author	**Year of Publication**	**LVAD Device model used, n (%)**					
		Heartmate I	Heartmate II	Heartware	Jarvik 2000	Novacor	Other
Shehab [16]	2018						75 (100%)
Yoshioka [15]	2017		240 (86%)	38 (14%)			
Takeda [14]	2013	168 (60%)	114 (40%)				
Charisopoulou [28]	2019						nr
Amsallem [27]	2018		107 (56%)	79 (41%)	1 (>1%)		5 (3%)
Kurihara [33]	2018		293 (77%)	86 (23%)			
Nitta [31]	2018						93 (100%)
Capoccia [44]	2017		12 (100%)				
Deschka [29]	2016		28 (nr)	28 (nr)			
Yost [32]	2016		212 (82%)	44 (18%)			
Patil [18]	2015		67 (44%)	62 (41%)	23 (15%)		
Cordtz [43]	2014		22 (100%)				
Wang [24]	2012		3 (15%)			4 (15%)	20 (70%)
Pettinari [34]	2012		38 (64%)				21 (36%)
Saito [35]	2012	9 (8%)	2 (2%)		8 (7%)	10 (9%)	79 (74%)
Drakos [30]	2010	25 (nr)				9 (nr)	141 (nr)
Kormos [1]	2010		484 (100%)				
Dang [19]	2006	108 (100%)					
Morgan [9]	2004	243 (100%)					
Number of studies (n)		5 /19	13/19	6/19	3/19	3/19	8/19
Number of patients (n)		553 (18%)	1622 (54%)	337 (11%)	32 (1%)	23 (>1%)	434 (15%)

List of LVAD devices names: HeartMate I and HeartMate II (Thoratec, Pleasanton, CA, USA); HeartWare (HeartWare, Framingham, MA, USA); Jarvik 2000 (Jarvik Heart, New York, NY, USA); Novacor (Worldheart, Salt Lake City, UT, USA) Other: Ventrassist (Ventracor Ltd, Chatswood, NSW, Australia); DuraHeart (Terumo Heart, Ann Arbor, MI, USA); EVAHEART (SunMedical, Shiga, Japan); HeartMate XVE/HeartMate VE/HeartMate 1000 IP (Thoratec, Pleasanton, CA, USA); HeartAssist5 (ReliantHeart, Houston, Tex, USA); Nipro-VAD and Toyobo, (Nipro Corporation, Osaka, Japan);

RVAD models used

Author	**Year of Publication**	**RVAD Device model used, n (%)**				
		Centrimag	Abiomed	Rotaflow	Thoractec PVAD	other
Schmack [41]	2018					11 (100%)
Khorsandi [17]	2017	34 (80%)	1 (2%)	8 (18%)		
Bhama [40]	2013	80 (100%)				
Shah [42]	2019					15 (100%)
Yoshioka [15]	2018	nr (nr)*				
Takeda [14]	2018	13 (32%)	25 (63%)		2 (5%)	
Samura [21]	2018					nr
Charisopoulou [28]	2017					nr

Amsallem [27]	2016					nr
Deschka [29]	2016			nr		nr
Yost [32]	2015	15 (100%)				
Patil [18]	2014	35 (100%)				
Wang [24]	2012	8 (30%)	3 (11%)		15 (56%)	1 (3%)
Kormos [1]	2012	14 (47%)	9 (30%)		4 (13.3%)	3 (10%)
Morgan [9]	2012		17 (100%)			
Number of studies (n)		7/15	5/15	1/15	3/15	4/15
Number of patients (n)		199 (62%)	55 (17%)	9 (3%)	21 (7%)	34 (11%)

*= "almost all the patients"

List of RVAD devices names: CentriMag (Thoratec, Pleasanton, CA, USA); Abiomed AB 5000 (Abiomed, Danvers, MA, USA); (Rotaflow; Maquet, Rastatt, Germany); Thoratec PVAD (Thoratec, Pleasanton, CA, USA). Other: Bio-Medicus Perfusion System (Medtronic Inc, Minneapolis, MN, USA), TandemLife TandemHeart plus ProtekDuo dual-lumen cannula (LivaNova, PLC, London, UK);

Supplementary Table 4: Pooled stroke rates and pooled transplantation rates

Pooled Stroke Rates

**Treatment**	**Study**	**Year of Publication**	**Stroke rate per 100 patients/year**	**I^2**	**tau^2**	**p**
LVAD	*Pettinari [34]*	2012	11 (5 to 27)			
LVAD	*Shehab [16]*	2018	13 (7 to 25)			
LVAD	*Kurihara [33]*	2018	20 (17 to 24)			
**Pooled LVAD**			**17 (12 to 24)**	**34.1**	**.04**	
LVAD&RVAD	*Shah [42]*	2018	33 (14 to 80)			
LVAD&RVAD	*Bhama [40]*	2018	11 (6 to 21)			
**Pooled LVAD&RVAD**			**18 (6 to 55)**	**75.8**	**.49**	
Pooled Overall			**17 (12 to 23)**	**46.8**	**.07**	**.91**

Pooled Transplantation Rates

**Treatment**	**Study**	**Year of Publication**	**Transplantation rate per 100 patients/year**	**I^2**	**tau^2**	**p**
LVAD	*Morgan [9]*	2004	50 (43 to 58)			
LVAD	*Dang [19]*	2006	62 (48 to 80)			
LVAD	*Pettinari [34]*	2012	64 (45 to 93)			
LVAD	*Saito [35]*	2012	17 (11 to 27)			
LVAD	*Patil [18]*	2015	12 (8 to 19)			
LVAD	*Deschka [29]*	2016	4 (1 to 17)			
LVAD	*Yoshioka [15]*	2017	22 (18 to 26)			
LVAD	*Shehab [16]*	2018	61 (46 to 82)			
**Pooled LVAD**			**31 (20 to 48)**	**94.7**	**.35**	
LVAD&RVAD	*Morgan [9]*	2004	6 (4 to 12)			
LVAD&RVAD	*Takeda [14]*	2013	7 (3 to 14)			
LVAD&RVAD	*Patil [18]*	2015	8 (4 to 17)			
LVAD&RVAD	*Deschka [29]*	2016	8 (2 to 24)			
LVAD&RVAD	*Yoshioka [15]*	2017	13 (6 to 27)			
LVAD&RVAD	*Shah [42]*	2018	100 (60 to 166)			
LVAD&RVAD	*Samura [21]*	2019	4 (2 to 10)			
**Pooled LVAD&RVAD**			**11 (4 to 29)**	**92.4**	**1.58**	
Pooled Overall			**19 (13 to 29)**	**94.5**	**.57**	**.055**

Pooled Transplantation Rates

Sensitivity analysis: Shah 2018 dropped

Treatment	Study	Year of Publication	Transplantation rate per 100 patients/year	I^2	tau^2	p
LVAD	*Morgan [9]*	2004	50 (43 to 58)			
LVAD	*Dang [19]*	2006	62 (48 to 80)			
LVAD	*Saito [35]*	2012	17 (11 to 27)			
LVAD	*Pettinari [34]*	2012	64 (45 to 93)			
LVAD	*Patil [18]*	2015	12 (8 to 19)			
LVAD	*Deschka [29]*	2016	4 (1 to 17)			
LVAD	*Yoshioka [15]*	2017	22 (18 to 26)			
LVAD	*Shehab [16]*	2018	61 (46 to 82)			
**Pooled LVAD**			31 (20 to 48)	94.7	.35	
LVAD&RVAD	*Morgan [9]*	2004	6 (4 to 12)			
LVAD&RVAD	*Takeda [14]*	2003	7 (3 to 14)			
LVAD&RVAD	*Patil [18]*	2015	8 (4 to 17)			
LVAD&RVAD	*Deschka [29]*	2016	8 (2 to 24)			
LVAD&RVAD	*Yoshioka [15]*	2017	13 (6 to 27)			
LVAD&RVAD	*Samura [21]*	2019	4 (2 to 10)			
**Pooled LVAD&RVAD**			**7 (5 to 10)**	**0**	**0**	
**Pooled Overall**			**17 (11 to 26)**	**94.5**	**.55**	**<0.001**

Supplementary Figure 1: Transplantation rates per 100 patient-years

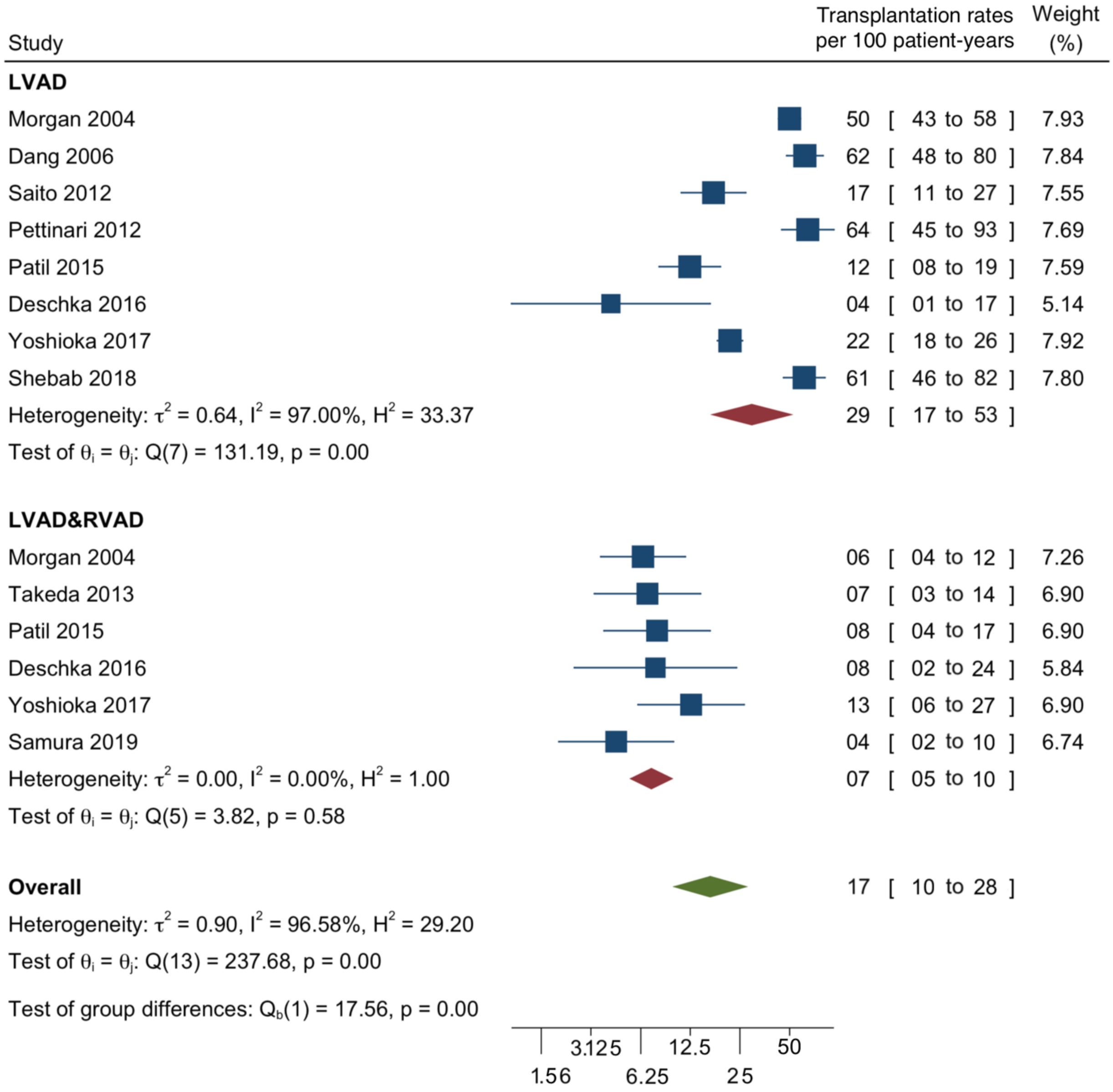

Supplementary Figure 2: Stroke rates per 100 patient-years

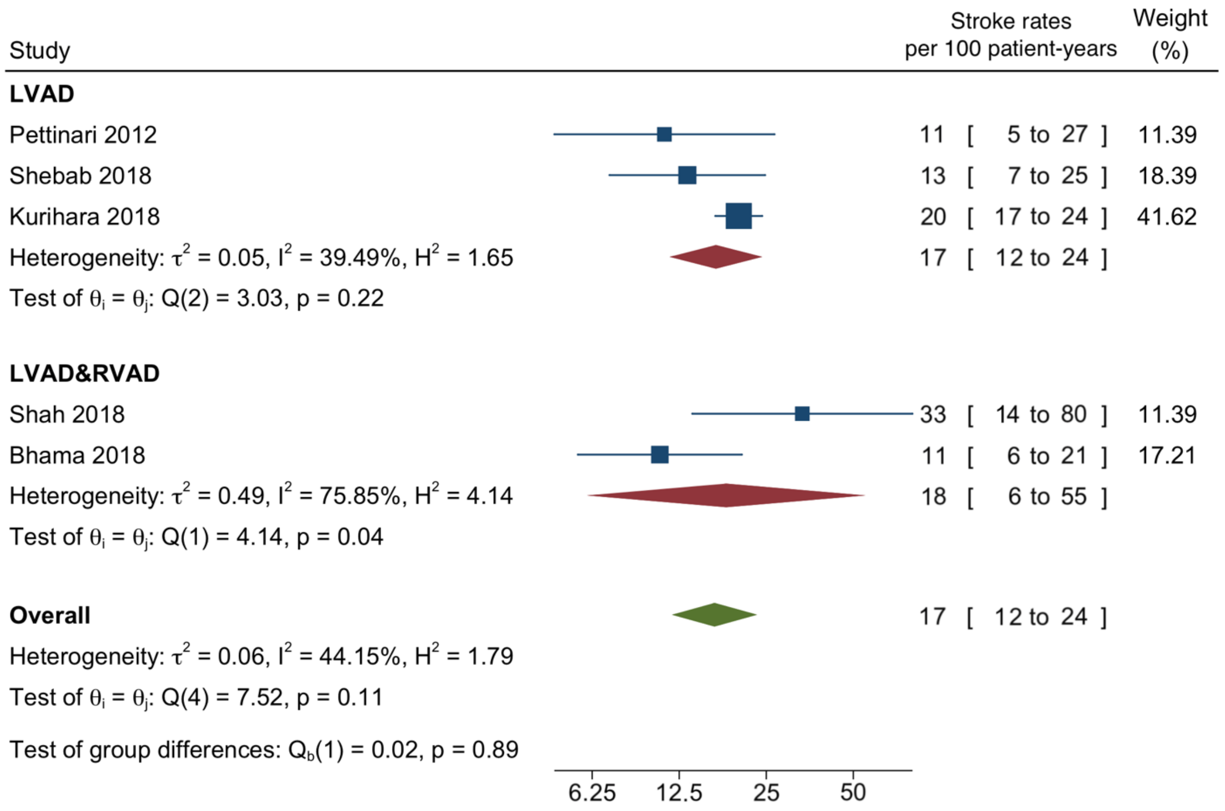

Supplement: sj-pdf-3-prf-10.1177_02676591211024817 – Supplemental material for Outcome of right ventricular assist device implantation following left ventricular assist device implantation: Systematic review and meta-analysis [file sj-pdf-3-prf-10.1177_02676591211024817.pdf]
